# Rationally Controlled Synthesis of CdSe_x_Te_1−x_ Alloy Nanocrystals and Their Application in Efficient Graded Bandgap Solar Cells

**DOI:** 10.3390/nano7110380

**Published:** 2017-11-08

**Authors:** Shiya Wen, Miaozi Li, Junyu Yang, Xianglin Mei, Bin Wu, Xiaolin Liu, Jingxuan Heng, Donghuan Qin, Lintao Hou, Wei Xu, Dan Wang

**Affiliations:** 1 School of Materials Science and Engineering, South China University of Technology, Guangzhou 510640, China; a740176840@163.com (S.W.); limz4994@sina.com (M.L.); depueedome@gmail.com (X.M.); wubin868888@163.com (B.W.); liulin9708@163.com (X.L.); hengjingxuan@163.com (J.H.); xuwei@scut.edu.cn (W.X.); wangdan@scut.edu.cn (D.W.); 2 Siyuan Laboratory, Guangzhou Key Laboratory of Vacuum Coating Technologies and New Energy Materials, Guangdong Provincial Key Laboratory of Optical Fiber Sensing and Communications, Department of Physics, Jinan University, Guangzhou 510632, China; junyuyang1992@163.com; 3 Institute of Polymer Optoelectronic Materials & Devices, State Key Laboratory of Luminescent Materials & Devices, South China University of Technology, Guangzhou 510640, China

**Keywords:** nanocrystal, solar cells, CdTe, graded bandgap

## Abstract

CdSe_x_Te_1−x_ semiconductor nanocrystals (NCs), being rod-shaped/irregular dot-shaped in morphology, have been fabricated via a simple hot-injection method. The NCs composition is well controlled through varying molar ratios of Se to Te precursors. Through changing the composition of the CdSe_x_Te_1−x_ NCs, the spectral absorption of the NC thin film between 570–800 nm is proved to be tunable. It is shown that the bandgap of homogeneously alloyed CdSe_x_Te_1−x_ active thin film is nonlinearly correlated with the different compositions, which is perceived as optical bowing. The solar cell devices based on CdSe_x_Te_1−x_ NCs with the structure of ITO/ZnO/CdSe/CdSe_x_Te_1−x_/MoO_x_/Au and the graded bandgap ITO/ZnO/CdSe(*w*/*o*)/CdSe_x_Te_1−x_/CdTe/MoO_x_/Au are systematically evaluated. It was found that the performance of solar cells degrades almost linearly with the increase of alloy NC film thickness with respect to ITO/ZnO/CdSe/CdSe_0.2_Te_0.8_/MoO_x_/Au. From another perspective, in terms of the graded bandgap structure of ITO/ZnO/CdSe/CdSe_x_Te_1−x_/CdTe/MoO_x_/Au, the performance is improved in contrast with its single-junction analogues. The graded bandgap structure is proved to be efficient when absorbing spectrum and the solar cells fabricated under the structure of ITO/ZnO/CdSe_0.8_Te_0.2_/CdSe_0.2_Te_0.8_/CdTe/MoO_x_/Au indicate power conversion efficiency (PCE) of 6.37%, a value among the highest for solution-processed inversely-structured CdSe_x_Te_1−x_ NC solar cells. As the NC solar cells are solution-processed under environmental conditions, they are promising for fabricating solar cells at low cost, roll by roll and in large area.

## 1. Introduction

Solution processed perovskite solar cells [1,2], polymer solar cells [3,4,5,6] and nanocrystal solar cells [7,8,9,10,11,12] have attracted much attention in recent year. Among which, CdTe-based thin film has been adopted in the photovoltaic market by virtue of its low-cost manufacturing and an ideal bandgap (~1.45 eV) for single-junction solar cells [13,14,15,16] lately, small-area CdTe solar cells have been proved efficient up to 22.1% [17] by First Solar. And yet the value remains below the Shockley limit for CdTe-based solar cells (~33%) [18]. Low *J*_sc_ (20–28 mA/cm^2^) evidently limits the performance of CdTe-solar cells due to the limited bandgap and bulk/interface recombination, while this value is ~40 mA/cm^2^ for single crystalline Si solar cells [19,20]. *N*-type CdS is typically adopted as the window layer for efficient CdTe-solar cells and another CdS_x_Te_1−x_ window layer is established at CdS/CdTe interfaces when depositing CdTe and heat-treating with CdCl_2_. However, it was reported that the formed CdSe_x_Te_1−x_ layer was not photoactive and no photo-currents were generated by the photons absorbed by the CdS_x_Te_1−x_ window layer [21]. Conversely, as Se into CdTe outstrips S in miscibility, a graded CdSe_x_Te_1−x_ layer can be formed easily when growing and post-treating the CdTe layer in the case of CdTe solar cells using CdSe as the window layer [22,23]. Under the “light bowing” effects, the CdSe_x_Te_1−x_ alloys exhibit a narrower bandgap than that of CdTe, elevating the external quantum efficiency (EQE) response to a longer wavelength [24,25,26]. Furthermore, CdSe is more completely consumed than other window layers, thus reducing the parasitic absorption and prolonging the short wavelength response of the device. Therefore, high device performance is anticipated with respect to CdTe-based solar cells with a CdSe-based window layer. Recently, Paudel [27] proved CdTe/CdSe-based solar cells following the structure of FTO/CdSe/CdTe/Cu/Au through adopting magnetron sputtering techniques with an efficiency of 15.2% coupled with a high *J*_sc_ of 26.3 mA/cm^2^. It was further optimized by Poplawsky et al. [28] through engineering the thickness of the CdSe film, fabricating an evidently high *J*_sc_ of 27.0 mA/cm^2^ (without a reflective layer) and PCE of 15.4%. It is indicated that CdSe_x_Te_1−x_ alloy is formed through diffusing Se or Te at the CdTe/CdSe interface in foregoing cases, which shall downgrade carrier mobility and not uniformize the CdSe_x_Te_1−x_ layers, consequently declining the device performance [28]. Based on the developing NC fabrication technology, high-quality CdSe_x_Te_1−x_ NCs composited differently can be easily controlled through varying the Se and Te content in the precursor before forming CdSe_x_Te_1−x_ alloy NC [29,30]. The performance of CdTe/CdSe NC solar cells is anticipated to be tailored by adopting a nanoscale CdSe_x_Te_1−x_ alloy layer. Furthermore, NC solar cells fabricated in the wake of solution processing are numerously advantageous, such as being low in cost, having a high chemical yield and having an easy-to-sinter property in virtue of the low melting point. There are several ways to introduce the CdSe_x_Te_1−x_ alloy NC as an active layer to attain illumination, which can be clarified from several perspectives. First and foremost, the CdTe and CdSe NCs are deposited in sequence and annealed at high temperature to form CdSe_x_Te_1−x_, consequently diffusing Se or Te at the CdTe/CdSe interface. Gur et al. [31] first demonstrated CdTe/CdSe NC solar cells with a PCE approaching 3%. Through adopting an inverted structure of ITO/CdSe/CdTe/Cr(*w*/*o*)/Au, the solar cells performance can be further optimized [32,33]. Recently, our research group illuminated a ZnO buffer layer between ITO and CdSe film, as high as 5.81% was attained in a configuration of ITO/ZnO/CdSe/CdTe/Au [34]. Another way to illuminate CdSe_x_Te_1−x_ as active layer is to sinter thin films containing mixtures of CdTe and CdSe NC. MacDonald et al. [35] and our group [36] generated a layer by layer NC sintering strategy to further optimize the quality of NC film with performance of 7.1%/6.25% attained in device structures of ITO/CdTe/CdSe_x_Te_1−x_/ZnO/Al and ITO/ZnO/CdSe/CdSe:CdTe/CdTe/Au. Unfortunately, although *J*_sc_ has been optimized, relative low *V*_oc_ (~0.6 V) is attained in the foregoing case. Alloy NC film can also be fabricated through directly depositing a solution containing CdSe_x_Te_1−x_ alloy NC onto the substrate. In this case, the active layer properties can be tuned through adjusting the composition of the alloy NC. Yang et al. exhibited a new device structure of ITO/TiO_2_/CdSe_x_Te_1−x_/MoO_x_/Au through adopting CdSe_x_Te_1−x_ alloy NC as active layer and a low PCE ~4% was attained because of low *J*_sc_ and *V*_oc_, which was primarily originated from large defect in active layer [37].

Here, we report on the synthesis of CdSe_x_Te_1−x_ alloy NC with entire compositional range via hot injection method. The transmission electron microscopy (TEM), energy-dispersive X-ray spectroscopy (EDS) and X-ray diffraction (XRD) are adopted to resolve morphology, structure and ascertain compositions. The composition of CdSe_x_Te_1−x_ alloy is ascertained to be well controlled through varying the ratio of the Se to Te ratio in the precursor, while the optical properties denote a non-linear response to Se content, known as the “light bowing” effect. Devices with graded bandgaps of ITO/ZnO/CdSe/CdSe_x_Te_1−x_/MoO_x_/Au and ITO/ZnO/CdSe(*w*/*o*)/CdSe_x_Te_1−x_/CdTe/MoO_x_/Au are fabricated through sintering layer-by-layer. The effects of the NC alloy composition, thickness and annealing temperature on the graded bandgap solar cells are ascertained and disserted specifically in line with experimental results. Our works here illuminates that high *J*_sc_ and *V*_oc_ can be attained simultaneously through tuning the device structure and device preparing process. By replacing the CdSe window layer as CdSe_0.8_Te_0.2_ alloy film, the FF, *V*_oc_ and PCE can be further optimized in the meantime and a PCE as high as 6.37% is attained for NC solar cells with a ITO/ZnO/CdSe_0.8_Te_0.2_/CdSe_0.2_Te_0.8_/CdTe/MoO_x_/Au structure, owing to the improved in-band alignment, film quality and low series resistance.

## 2. Experiment

CdSe_x_Te_1−x_ NCs were prepared under environmental conditions. Trioctylphosphine selenide (TOP-Se) and TOP-Te precursor were prepared through dissolving 8 mmol Se and 8 mmol Te into 10 mL TOP solvent, respectively. When typically preparing CdSe_0.2_Te_0.8_ NCs, 0.2 mL TOP-Se was mixed with 0.8 mL TOP-Te in a fume hood under environmental conditions. The mixed TOP-Se/Te solution was then stirred for six hours to attain a clear and homogeneous solution. In a three-necked flask equipped with a thermometer, 905.6 mg (1.6 mmol) of cadmium myristate, 114 mg myristate acid (0.5 mmol) and 2.40 g trioctylphosphine oxide (TOPO) were added. The mixtures were heated up to 240 °C under N_2_ flow. When heating, cadmium myristate was dissolved progressively and a faint yellow homogeneous solution was generated. The mixture was kept at this temperature for 10 min and then 1 mL TOPSe-Te solution was quickly injected into the reaction flask and the whole reaction lasted for 30 min. To fabricate alloy NCs with compositions, TOP-Se/TOP-Te of different ratios was conducted while the other conditions remained unchanged. After the reaction was cooled to room temperature, the mixture was washed three times with methanol/toluene and centrifuged. The final outcomes were refluxed in pyridine for 24 h and centrifuged with n-hexane. The CdSe_x_Te_1−x_ NCs products were re-dispersed into pyridine/1-propanol (1:1 *v*/*v*) with a concentration of 45 mg/mL. The ZnO precursor, CdSe NCs and CdTe NCs were prepared following our previous works [38,39]. The NC graded bandgap solar cells were fabricated following a layer-by-layer solution process, as reported in the previous work [33]. It is noteworthy that the concentrations of all the alloy NC solutions are attained as 45 mg/mL (the concentration of CdSe NC solution is 30 mg/mL), which permits similar thicknesses for one layer of NC thin film. After depositing the NC active layer, 8 nm MoO_x_ and 100 nm Au were evaporated through a mask at a pressure below 10^−5^ Torr, achieving an active area of 0.16 cm^2^.

## 3. Results and Discussion

**Figure 1:**
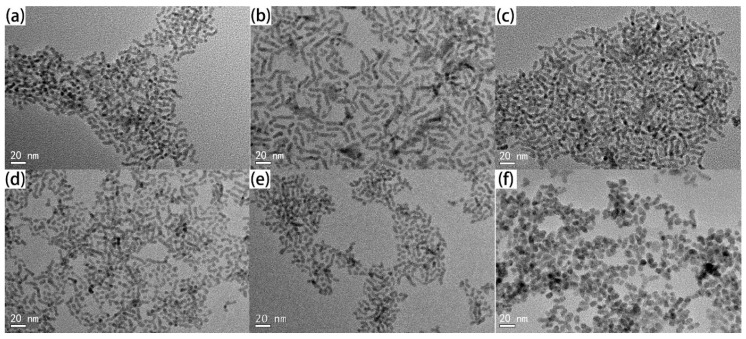
TEM images of CdSe_x_Te_1−x_ alloy NCs with different composition: (**a**) *x* = 0, (**b**) *x* = 0.1, (**c**) *x* = 0.2, (**d**) *x* = 0.4, (**e**) *x* = 0.6 and (**f**) *x* = 0.

**Figure 2:**
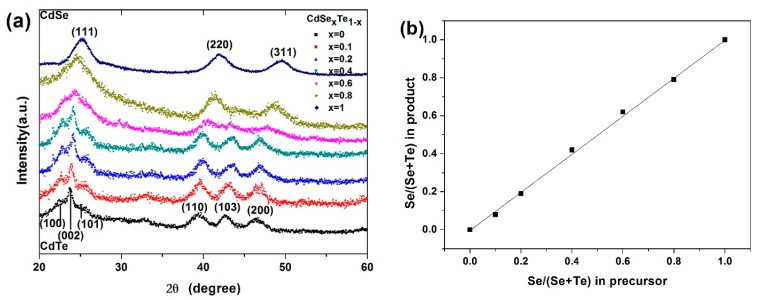
(**a**) The XRD pattern of the CdSe_x_Te_1−x_ NCs with different composition and (**b**) the EDS results for CdSe_x_Te_1−x_ NCs with different composition.

CdSe_x_Te_1−x_ materials composited differently are compounded through injecting hybrid TOP-Se/TOP-Te into the Cd^2+^ carboxylic precursor and adopting TOPO as the coordinate solvent at a moderate temperature of 240 °C. It is ascertained that the CdSe_x_Te_1−x_ alloy NCs indicate a morphological rod shape when *x* < 0.8 (Figure 1a–f), while the irregular dot shape is attained for *x* = 0.8 samples, which conforms to our previous report for CdTe/CdSe NCs compounded under the same condition [38,39]. In terms of CdSe or CdTe NC compounded through adopting coordinating organic solvents (TOPO) and carboxyl acid as ligands, elongated NCs were attained under the confined monomer concentration, which had been extensively ascertained before [40,41,42]. The average arm length/arm diameters for *x* = 0, 0.1, 0.2, 0.4, 0.6, 0.8 include 17.5 nm/5.8 nm, 15.2 nm/5.5 nm, 14.9 nm/5.2 nm, 13.2 nm/4.3 nm, 12.9 nm/4.9 nm and 11.4 nm/7.9 nm, respectively. Evidently, the diameter of alloy NC is approximately 5 nm while the length decreases as the Se content increases (*x* < 0.8). The NC indicates an irregular dot shape with high Se content, similar to that reported of pure CdSe NC [38]. More homogeneous NC is attained with low Se content, while a large size distribution is detected with high Se content. This can be illuminated as follows: The CdTe seeds outstrip CdSe seeds in formation speed, consequently accelerating growth of morphologically elongate NC in this case [39]. Conversely, low NC seed growth rates in the case of high Se content will result in large alloy NCs, which is confirmed in previous reports [39]. The crystal structures of the CdSe_x_Te_1−x_ NCs are further characterized by powder XRD. The XRD pattern (Figure 2a) of the CdSe_x_Te_1−x_ NCs with different Se contents bespeak similar diffraction peaks which can be indexed as (100), (002), (101), (110), (103) and (200) or (111), (220) and (311) facets of wurtzite CdTe/zinc blend CdSe. It is ascertained that the diffraction peaks shall be progressively converted into larger angles with the rising Se content. In contrast with pure CdSe or CdTe NC, the XRD patterns of CdSe_x_Te_1−x_ alloy NC denotes an evident size-broadening effect as the lattice constant varies. No phase separation or separated nucleation of CdTe/CdSe in the CdSe_x_Te_1−x_ is detected, indicative of the formation of a homogeneous alloy. The alloy NC composition is dependent on energy disperse spectroscopy (EDS) measurements (all of the alloy NC samples are prepared by deposit 240 nm NC film on ITO substrate and annealing at 350 °C for 30 min), as exhibited in Figure 2b (EDS image for CdSe_0.2_Te_0.8_ and CdSe_0.4_Te_0.6_ NC is presented in Figure S1). It is noteworthy that the relative amount of Se to (Se + Te) in the alloy NC thin film is basically the same as that in the precursor solution, illuminating that all the Se and Te have been transformed into CdSe_x_Te_1−x_ as the Cd precursor is excessive in this reaction (with Cd/(Se + Te) = 2:1 in all reactions).

**Figure 3:**
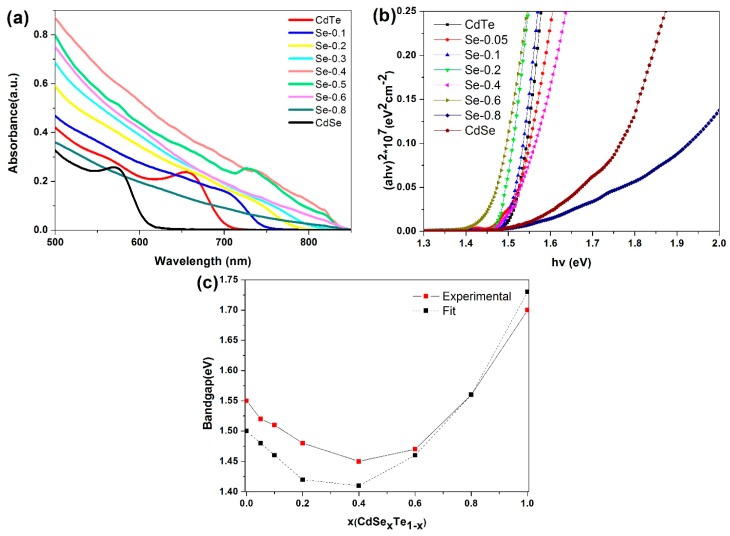
(**a**) The absorption patterns of the CdSe_x_Te_1−x_ NCs with different Se content; (**b**) the plots of (аhv)^2^ versus photon energy of the CdSe_x_Te_1−x_ NCs thin film with different Se content; and (**c**) the bandgap value of the CdSe_x_Te_1−x_ NCs thin film with different Se content.

The absorption peak for pure CdSe and CdTe is 570 nm and 660 nm, respectively, as presented in Figure 3a. Evidently, the absorption peak is sharp for pure CdSe or CdTe (*x* = 1 or *x* = 0), indicating the formation of NCs with homogeneous size and narrow in size distribution. For the ternary alloy CdSe_x_Te_1−x_ NCs, the variation in absorption edge is ascertained to be composited differently. For NCs containing a low Se content, the absorption edge can be differentiated. Conversely, no peaks are distinguished for NC with a high Se content and a long tail in the absorption spectrum because of the extensive size distribution and the alloy structure. In contrast with the absorption edge of CdTe NCs, the absorption edge shifted from 660 to 780 ± 30 nm in the case of *x* = 0.1 to 0.6. The spectral shifts demonstrated in Figure 3a are mainly caused by the nonlinear relationship between the composition and the bandgap of CdSe_x_Te_1−x_ alloy, which refers to “light bowing” effects [43,44]. As the NC thin film will recover its bulk bandgap after annealing/chemical treatment, the optical properties of the NC alloy thin film adopted as the active layer will determine the performance of the solar cells. Therefore, to ascertain how the bandgap and composition are related to each other, NC alloy thin film structured by ITO/NC are prepared. All the NC thin films are prepared through depositing three layers (~240 nm) of NC solution onto the ITO substrate and annealing at 350 °C for 30 min. It is ascertained for all compositions the plots of (аhv)^2^ versus the photon energy have a linear onset, indicative of a direct bandgap (Figure 3b). We can determine the optical bandgap of alloy NC thin films by extrapolating the linear region of this plot to the *x*-axis. As summarized in Figure 3c, the bandgap value between 1.45 to 1.70 eV is attained with different Se content. For CdSe_x_Te_1−x_ alloy semiconductors, the bandgap vs composition can be conventionally compatible with a bowing formula [44]. *E*_g_(*x*) = (1 − *x*)*E*_g_(CdSe) + *xE*_g_(CdTe) − b*x*(1 − *x*), where b = 0.75 is the optical bowing coefficient and the values of *E*_g_(CdSe) and *E*_g_(CdTe) are 1.73 eV and 1.50 eV, respectively. The experiment results conform well to compatible results at high Se content (*x* > 0.6), with a slight shifting at low Se content (*x* = 0.1 to 0.4).

To ascertain the charge transport properties of alloy NC with composition, we fabricate hole-only devices with a structure of ITO/CdSe_x_Te_1−x_ (160 nm)/MoO_x_(5 nm)/Au(70 nm). All alloy NC thin films are annealed at 350 °C with CdCl_2_ treatment for 30 min. The hole carrier mobility of CdSe_x_Te_1−x_ NC thin film is ascertained via the space-charge-limited-current (SCLC) method. The carrier mobility is attained in line with the following equation [45]:

$$J=\frac{9}{8}\frac{\varepsilon_{0}\varepsilon_{r}\mu_{p}{\left(V-V_{bi}-V_{s}\right)}^{2}}{L^{3}}$$

where $\varepsilon_{0}$ is the permittivity of free space, $\varepsilon_{r}$ refers to the relative dielectric constant of CdSe_x_Te_1−x_, **L** is the thickness of alloy NC, **μ_p_** is the hole mobility, ***V*** is the applied voltage and ***V*_s_** is the voltage drop due to contact resistance, while ***V*_bi_** is the built-in voltage. As exhibited in Figure S2 and Table 1, the mobility for *x* = 0, 0.1, 0.2, 0.4, 0.6, 0.8 are 2.02 × 10^−4^, 3.48 × 10^−4^, 4.05 × 10^−4^, 4.34 × 10^−4^ and 3.07 × 10^−4^ cm^2^/Vs, respectively. The value of ternary alloy NC is basically two orders higher than those ever reported [37].

**Figure 4:**
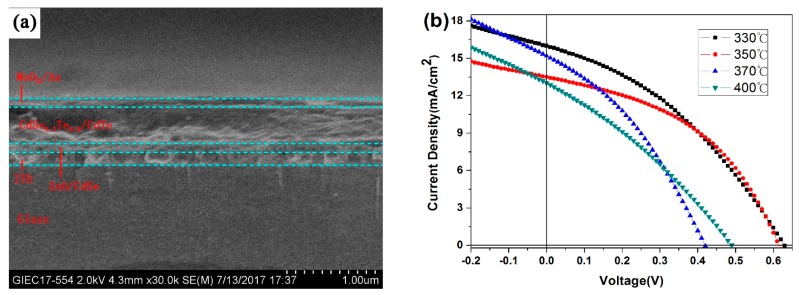
(**a**) Cross-section SEM image of the glass/ITO/ ZnO/CdSe/CdSe_0.2_Te_0.8_/CdTe/Au/MoO_x_ device and (**b**) *J*-*V* characteristics of ITO/ZnO/CdSe/CdSe_0.2_Te_0.8_/CdTe/Au/MoO_x_ devices with different annealing temperatures under light.

CdSe NC had been proved to be a good partner for CdTe NC solar cells in our previous report [34]. To ascertain the performance of solar cells with alloy NC as active layer, the CdSe_0.2_Te_0.8_ NC film is adopted as the active layer and devices with an inverted structure of ITO/ZnO (40 nm)/CdSe (30 nm)/CdSe_0.2_Te_0.8_ (400 nm)/MoO_x_ (8 nm)/Au (80 nm) are fabricated through depositing five NC layers on CdSe film. The cross-section SEM image of NC device is exhibited in Figure 4a. The thickness of active layer is approximately 400 nm, similar to that reported for CdTe NC device with similar processing conditions [34]. It is well known that an appropriate annealing temperature is necessary for high-quality NCs thin films [32,33]. An elevated temperature shall increase the crystal size, eliminate interface defects and optimize the carrier mobility. The *J*-*V* curves for alloy NC solar cells with different annealing temperatures are exhibited in Figure 4b and the photovoltaic parameters are summarized in Table 2. The devices annealing at a moderate temperature of 350 °C exhibit a short circuit current density (*J*_sc_) of 16.04 mA/cm^2^, an open circuit voltage (*V*_oc_) of 0.63 V, a fill factor (FF) of 36.68% and a PCE of 3.71%. It is ascertained that device performance degrades with the increase of temperature, which is primarily ascribed to the reduced *V*_oc_ (below 0.5 V). For CdTe NC-based solar cells, high annealing temperatures shall partially resolve the NC film and make a device shunt, which has been confirmed previously [46]. For this reason, we adopt 350 °C as the annealing temperature for all the devices discussed below. Devices fabricated using ternary alloy NCs as the active layer generally have a lower PCE around 2−4% (see Table 2), while this value is ~6% for CdTe/CdSe solar cells, due to lower *J*_sc_ and FF, similar results is also attained by Yang et al. [37]. The device technology is not further tuned in this paper in terms of these structures and this paper asserts there may be room for further improvement.

**Figure 5:**
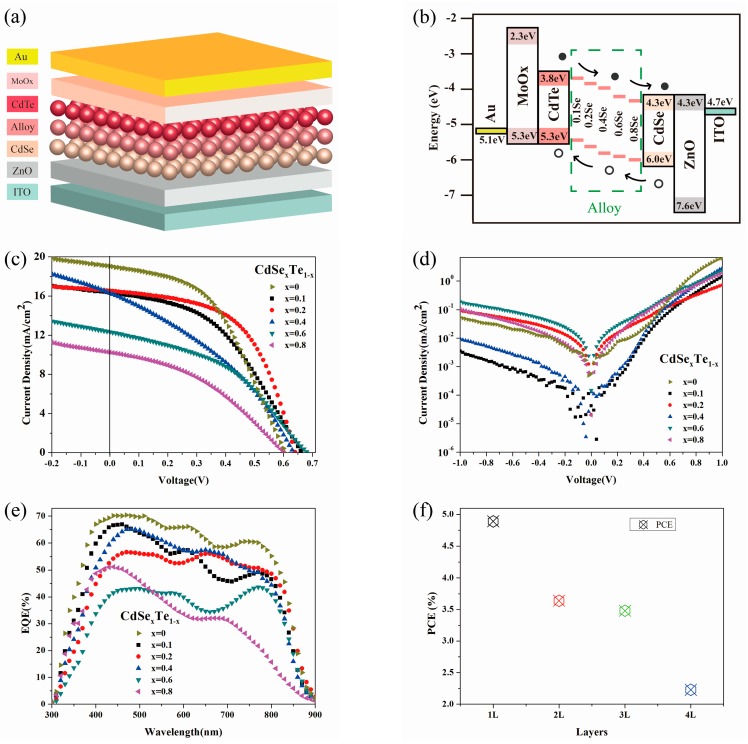
(**a**) Schematic of the vertically-graded bandgap structure of ITO/ZnO/CdSe/CdSe_x_Te_1−x_/CdTe/MoO_x_/Au, (**b**) energy diagram of the above devices, *J*-*V* characteristics of the above devices (**c**) under light and (**d**) dark, (**e**) EQE properties of the above devices, (**f**) the PCE of devices with different thickness of CdSe_0.2_Te_0.8_ alloy NC films (device structure: ITO/ZnO/CdSe/CdSe_0.2_Te_0.8_/CdTe/MoO_x_/Au).

To surmount the drawback of low *J*_sc_ and FF attained in NC solar cell devices with a single-alloy active layer, the vertically-graded bandgap is formed by using the layer-by-layer solution processed method. In the case of devices with single bandgaps as the active layer, they can absorb only the photons with energy larger than, or equal to, the bandgap of the material. Conversely, in devices with two or more semiconductor active layers, the absorption range is extended and most of the photons available within the solar spectrum can be attained through designing an optimized device structure [47]. Otherwise, the slope of the built-in electric field shall decrease the carrier recombination and increase the carrier collection efficiency. This new design for CdTe-graded bandgap devices has been experimentally tested using a structure of glass/FTO/n-ZnS/n-CdS/n-CdTe/Au recently [48,49]. Here, the graded bandgap devices with a structure of ITO/ZnO/CdSe(*w*/*o*)/CdSe_x_Te_1−x_/CdTe/MoO_x_/Au is presented in Figure 5a, while the band alignment is presented in Figure 5b (attained in line with previous report [35,37]. The CdSe_x_Te_1−x_ alloy NC active layers consisting of one or more bandgaps are prepared through depositing layer by layer. The optimized active layer is constituted by one layer of CdSe NC (~30 nm), one layer of CdSe_x_Te_1−x_ NC (~80 nm) and four layers of CdTe NCs (~320 nm). The introduction of CdSe_x_Te_1−x_ alloy NC film is anticipated to extend the optical response to longer wavelengths. The thickness of the CdSe_x_Te_1−x_ NCs/CdTe NCs shall evidently exert influence on the band alignment and spectrum response of NC solar cells. Therefore, the influence of thickness of alloy NC film on device performance is ascertained with the CdSe_0.2_Te_0.8_ NCs. It is attained that the device performance degrades with the increase of alloy NC thickness (Figure 5f). The PCE for one layer alloy NC film is 4.89%, while this value is 3–4% for thick alloy NC films, as exhibited in Figure 5f and Table 3. The *J*-*V* curves for graded bandgap devices with one layer of alloy NC film are illustrated in Figure 5a and the photovoltaic parameters are detailed in Table 3. It is attained that the *V*_oc_ of all the devices is higher than 0.6 V and 0.67 V/0.66 V is attained in the case of CdSe_0.6_Te_0.4_/CdSe_0.1_Te_0.9_, while this value is only 0.61 V for pure CdTe NC active layer. The champion device is attained by employing a structure of ITO/ZnO/CdSe/CdSe_0.2_Te_0.8_/CdTe/MoO_x_/Au. The device exhibits a short circuit current density (*J*_sc_) of 16.54 mA/cm^2^, an open circuit voltage (*V*_oc_) of 0.63 V, a fill factor (FF) of 54.93% and a high PCE of 5.75% (the values of other three devices in this thin film are 5.56%, 5.62% and 5.68% respectively). This value is higher than the controlled devices without alloy an NC layer (with PCE of 5.45% for devices with the structure of ITO/ZnO/CdSe/CdTe/MoO_x_/Au). From the *J*-*V* curve (Figure 5c) and Table 2, it is determined that better device performance is attained through adopting a low Se content as the alloy active layer, while device performance degrades with the rise of Se content, primarily suffering from low *J*_sc_ and fill factor. It should be pointed out that the devices prepared under this way are homogeneous with efficiency difference less than 10% in each single film (For each type, over 20 devices are made from 4 individual films. The dark *J*-*V* curves for devices with different alloy NC films are detailed in Figure 5d. The leakage current is increased almost linearly with the Se content increase in CdSe_x_Te_1−x_ alloy. The corresponding external quantum efficiency (EQE) for devices with different alloy NC films is presented in Figure 5e. The decreasing EQE response for alloy NCs with increased Se content matches well with our *J*_sc_ result obtained from *J*-*V* curve measurement. As the carrier mobility of CdSe_x_Te_1−x_ NC alloy has a similar value, the change in device performance with different NC alloy active layers cannot be attributed to the change in carrier transfer properties alone. We speculate that, in the case of CdSe_x_Te_1−x_ with low Se content, the lattice constant is near the value of CdTe and the interface (CdTe/CdSe_x_Te_1−x_) defects are low after annealing. The decreased defects between the alloy NC layer and the CdTe NC layer shall optimize carrier departure and collection efficiency. Therefore, high device performance is obtained in the case of using an alloy NC layer with low Se content. On the contrary, large lattice mismatch between the alloy NC layer with high Se content and the CdTe NC film shall reduce carrier collection efficiency and device performance.

**Figure 6:**
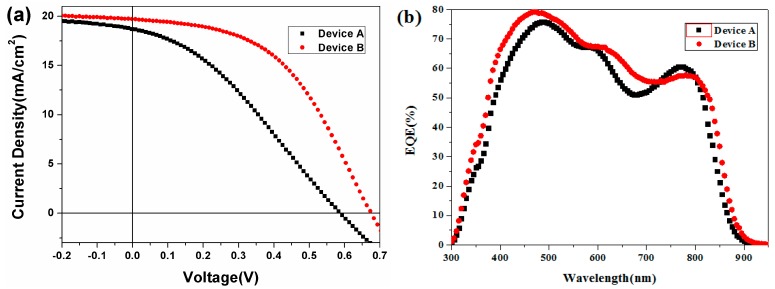
(**a**) *J*-*V* characteristics of Device A (ITO/ZnO/CdSe_0.2_Te_0.8_/CdTe/MoO_x_/Au, black) and Device B (ITO/ZnO/CdSe_0.8_Te_0.2_/CdSe_0.2_Te_0.8_/CdTe/MoO_x_/Au, red) under light (inset shows the *J-V* under dark) and (**b**) the corresponding EQE properties of these devices.

To further eliminate the interface mismatch between CdSe_x_Te_1−x_ NC alloy active layers, more CdSe_x_Te_1−x_ layers shall be adopted in the graded bandgap system. From another perspective, the existing large bandgap CdSe film shall accelerate the parasitic absorption and degrade the device performance. Here, for the sake of further tuning the ITO/ZnO/CdSe/CdSe_0.2_Te_0.8_/CdTe/MoO_x_/Au device performance, we selected CdSe_0.8_Te_0.2_ as the front contact for ZnO and designed a new device structure of ITO/ZnO/CdSe_0.8_Te_0.2_/CdSe_0.2_Te_0.8_/CdTe/MoO*_x_*/Au without the CdSe layer, which consists of one layer of CdSe_0.8_Te_0.2_, one layer of CdSe_0.2_Te_0.8_ and three layers of CdTe NC. For comparing, controlled devices without CdSe_0.8_Te_0.2_ (ITO/ZnO/CdSe_0.2_Te_0.8_/CdTe/MoO_x_/Au) are also prepared (Figure 6a and Table 4). The graded bandgap device without CdSe NC film is ascertained to display the following figures of merit: *J*_sc_ of 19.70 mA/cm^2^, *V*_oc_ of 0.67 V, a FF of 48.25% and a high PCE of 6.37%, which is among the highest PCE ever reported for CdSe_x_Te_1−x_ NC alloy solar cells (Table 4). Conversely, the device without the CdSe_0.8_Te_0.2_ layer indicate a *J*_sc_ of 18.59 mA/cm^2^, *V*_oc_ of 0.59 V, FF of 32.56% and a low PCE of 4.03%. The EQE curves NC devices denotes a prominent response confined into the whole range of 400–900 nm, when they are integrated, current density of 19.62 mA/cm^2^ and 18.40 mA/cm^2^ are predicted, respectively, which is consistent with our AM1.5 G measurements (Figure 6b). It is noteworthy that the FF of the device remains under 0.5, well lower than the relative high value (around 0.7) for CdTe NC solar cells with the normal structure of ITO/CdTe/ZnO/Al [50]. It is highlighted that the high FF obtained in normal device is based on light/current treatment. However, current/light soaking has no effects on the graded bandgap devices with inverted structure in our case, which is also ascertained by Panthani et al. [51]. As the MoO_x_ could form an ohmic contact for CdTe-based solar cells, the back contact shall not eliminate the fill factor. We speculated that the lattice mismatch between NC alloy layers resulted in an increase of *R*_s_ and a low FF is expected in this case. It is worth to point out that the device is very stale when keeping under room light condition, less than 5% degrade is found over 30 days. We believe that by further optimizing the device fabrication process/device structure, such as using more alloy NC active layers, carefully controlling the annealing strategy, or using a more suitable back contact, device performance with PCE up to 10% will be obtained.

## 4. Conclusions

In summary, CdSe_x_Te_1−x_ NCs composited differently have been fabricated successfully by controlling the mole ratio between the TOP-Se precursor and TOP-Te precursor under elevated temperature. NC with high Se content indicates a larger size and shorter length. The light bowing effect is attained in the NC solution and NC thin film. Through adopting these alloy NCs, the solar cell devices are successfully fabricated with a graded bandgap structure of ITO/ZnO/CdSe(*w*/*o*)/CdSe_x_Te_1−x_/CdTe/MoO_x_/Au. The performance of the graded bandgap NC solar cells is primarily influenced by the lattice matching degree between the alloy NC layer, band alignment and the mobility of the NC thin film. The introduced alloy active layer can extend the spectrum response to longer wavelengths due to the low bandgap nature of the alloy NC thin film. Through adopting a structure of ITO/ZnO/CdSe_0.8_Te_0.2_/CdSe_0.2_Te_0.8_/CdTe/MoO_x_/Au, high *V*_oc_ and high *J*_sc_ are effected, attaining a power conversion efficiency up to 6.37%. This work also indicates routes to further tune performance by controlling the annealing strategies and the design of new graded bandgap device architectures.

## Figures and Tables

**Table 1:** Hole mobility of CdSe_x_Te_1−x_ NC thin films.

Sample	Hole Mobility (cm^2^v^−1^s^−1^)
CdTe	2.02 × 10^−4^
CdSe_0.1_Te_0.9_	2.20 × 10^−4^
CdSe_0.2_Te_0.8_	3.48 × 10^−4^
CdSe_0.4_Te_0.6_	4.05 × 10^−4^
CdSe_0.6_Te_0.4_	4.34 × 10^−4^
CdSe_0.8_Te_0.2_	3.07 × 10^−4^

**Table 2:** Summarized performances of CdSe_0.2_Te_0.8_ NC solar cells with different annealing temperatures (Figure 4b).

Annealing Temperature (°C)	*V*_oc_ (V)	*J*_sc_ (mA/cm^2^)	FF (%)	PCE (%)
330	0.62	13.55	43.67	3.67
350	0.63	16.04	36.68	3.71
370	0.42	15.23	35.22	2.25
400	0.49	13.06	31.11	1.99

**Table 3:** Summarized performances of vertically-graded bandgap NC solar cells (Figure 5).

Device	*V*_oc_ (V)	*J*_sc_ (mA/cm^2^)	FF (%)	PCE (%)	$R_{s}$ (Ω·cm^2^)	$R_{sh}$ (Ω·cm^2^)
ITO/ZnO/CdSe/CdSe_0.05_Te_0.95_/CdTe/MoO_x_/Au	0.69	11.31	32.77	2.56	28.32	227.97
ITO/ZnO/CdSe/CdSe_0.1_ Te_0.9_/CdTe/MoO_x_/Au	0.66	16.32	44.10	4.77	22.15	244.44
ITO/ZnO/CdSe/CdSe_0.2_ Te_0.8_/CdTe/MoO_x_/Au	0.63	16.54	54.93	5.75	9.24	442.68
ITO/ZnO/CdSe/CdSe_0.4_ Te_0.6_/CdTe/MoO_x_/Au	0.63	16.32	35.26	3.64	17.99	87.36
ITO/ZnO/CdSe/CdSe_0.6_ Te_0.4_/CdTe/MoO_x_/Au	0.67	12.34	42.28	3.51	33.89	166.83
ITO/ZnO/CdSe/CdSe_0.8_ Te_0.2_/CdTe/MoO_x_/Au	0.60	10.26	38.78	2.39	33.30	226.21
ITO/ZnO/CdSe/CdTe/MoO_x_/Au	0.61	19.05	46.91	5.45	16.20	236.09

**Table 4:** Summarized performances of Device A and Device B (Figure 6).

Device	*V*_oc_ (V)	*J*_sc_ (mA/cm^2^)	FF (%)	PCE (%)	$R_{s}$ (Ω·cm^2^)	$R_{sh}$ (Ω·cm^2^)
ITO/ZnO/CdSe_0.2_Te_0.8_/CdTe/MoO_x_/Au	0.59	18.59	32.56	4.03	25.94	147.68
ITO/ZnO/CdSe_0.8_Te_0.2_/CdSe_0.2_Te_0.8_/CdTe/MoO_x_/Au	0.67	19.70	48.25	6.37	15.00	422.13

## Supplementary Materials

The following are available online at www.mdpi.com/2079-4991/7/11/380/s1, Figure S1: EDS of alloy NC (a) CdSe_0.2_Te_0.8_ NC and (b) CdSe_0.4_Te_0.6_ NC, Figure S2: Linearly fitting SCLC measurements of CdSe_x_Te_1-x_ alloy NC thin films with composition (x) (a) 0, (b) 0.1, (c) 0.2, (d) 0.4, (e) 0.6, (f) 0.8, where ε_0_ = 8.85 × 10^−12^, ε_r_ = 10, L = 160 nm, V_bi_ + V_s_ = 0.3 V.
