# Supplementary material for: Rationally Controlled Synthesis of CdSexTe1−x Alloy Nanocrystals and Their Application in Efficient Graded Bandgap Solar Cells
Source: Nanomaterials (Basel). 2017 Nov 8;7(11):380. doi: 10.3390/nano7110380 (PMC5707597; doi:10.3390/nano7110380)
Supplement: Supplementary file 1 [file nanomaterials-07-00380-s001.pdf]

Rationally Controlled Synthesis of $CdSe_xTe_{1-x}$ Alloy Nanocrystals and Their Application in Efficient Graded Bandgap Solar Cells

Shiya Wen [1,†], Miaozi Li [1,†], Junyu Yang [2], Xianglin Mei [1], Bin Wu [1], Xiaolin Liu [1], Jingxuan Heng [1], Donghuan Qin [3,*], Lintao Hou [2,*], Wei Xu [3] and Dan Wang [3]

Electronic Supplementary Material

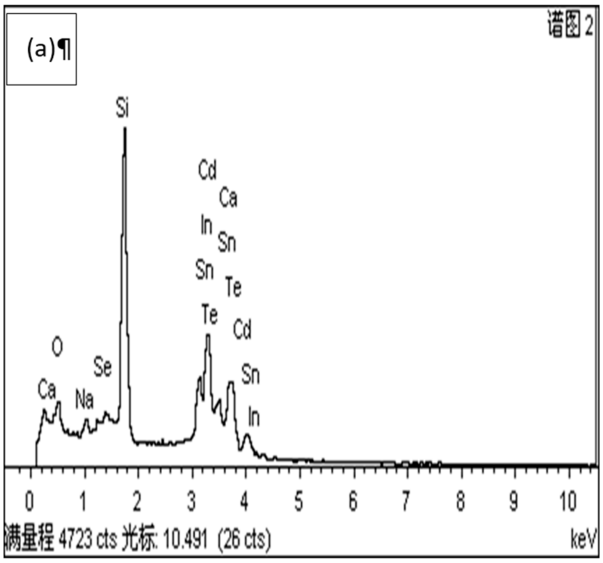

element	Weight percentage	Atomic percentage
O K	15.48	38.15
Na K	2.60	4.46
Si K	24.34	34.17
Ca K	5.53	5.44
Se L	1.58	0.79
Cd L	12.60	4.42
In L	22.92	7.87
Sn L	4.01	1.33
Te L	10.93	3.38
Total	100.00	100
Se / (Se+ Te)=0.19		

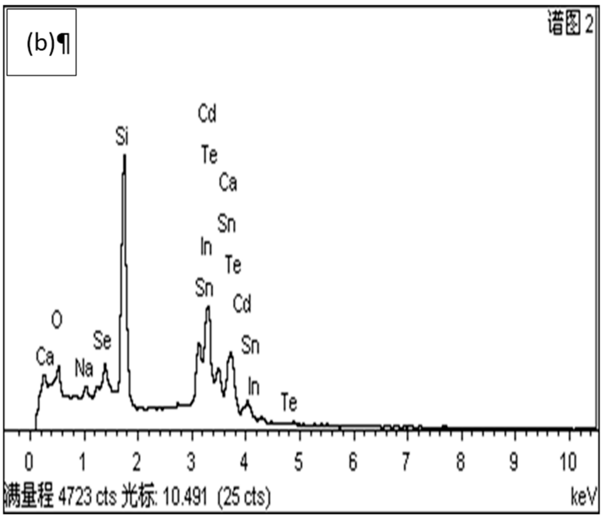

element	Weight percentage	Atomic percentage
O K	15.96	40.35
Na K	2.28	4.02
Si K	21.31	30.68
Ca K	4.94	4.99
Se L	4.62	2.36
Cd L	13.87	4.99
In L	22.16	7.81
Sn L	4.48	1.53
Te L	10.37	3.29
Total	100.00	100
Se / (Se+Te)=0.42		

Figure S1. EDS of alloy NC (**a**) $CdSe_{0.2}Te_{0.8}$ NC and (**b**) $CdSe_{0.4}Te_{0.6}$ NC

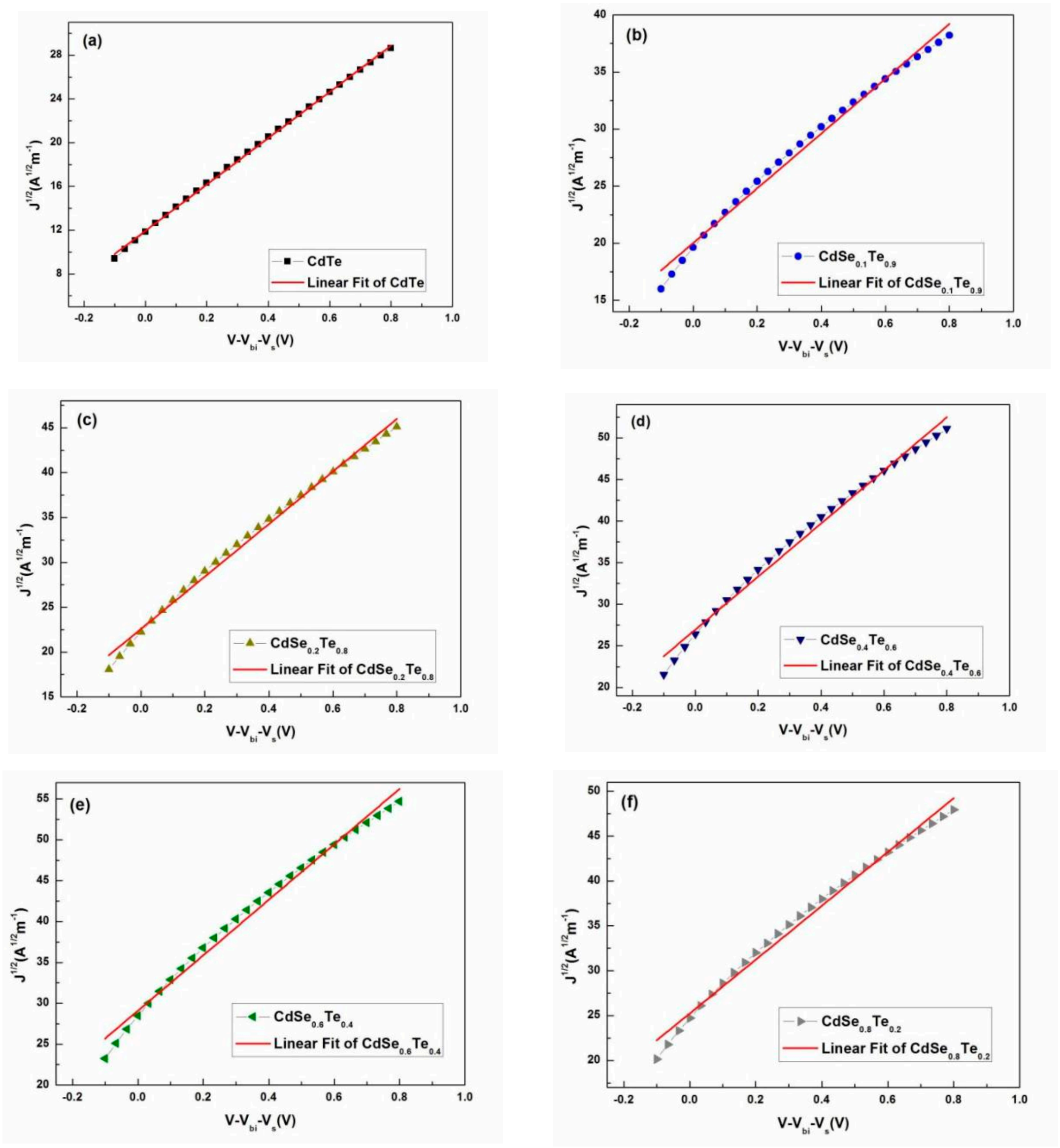

Figure S2. Linearly fitting SCLC measurements of $CdSe_xTe_{1-x}$ alloy NC thin films with composition (x) (**a**) 0, (**b**)0.1, (**c**) 0.2, (**d**) 0.4, (**e**) 0.6, (**f**) 0.8, where $\varepsilon_0 = 8.85\times10^{-12}$, $\varepsilon_r = 10$, L = 160 nm, $V_{bi} + V_s = 0.3$ V.
